# Associations between smoke exposure and osteoporosis or osteopenia in a US NHANES population of elderly individuals

**DOI:** 10.3389/fendo.2023.1074574

**Published:** 2023-02-03

**Authors:** Wenyuan Hou, Shaoqi Chen, Caiyu Zhu, Yifan Gu, Lei Zhu, Zhengxin Zhou

**Affiliations:** ^1^ The First Affiliated Hospital, Anhui University of Chinese Medicine, Hefei, China; ^2^ Department of Graduate School, Anhui University of Chinese Medicine, Hefei, China

**Keywords:** nonlinear associations, osteoporosis, osteopenia, serum cotinine, smoking, NHANES

## Abstract

**Background:**

Tobacco exposure is considered to be a risk factor for reduced bone mineral density (BMD), which may result in osteopenia. Cotinine, a metabolite of nicotine, is commonly utilized as a marker of tobacco exposure. Nevertheless, there are limited clinical data on the associations between osteoporosis (OP) or osteopenia and smoking status or serum cotinine level.

**Methods:**

We thoroughly examined the NHANES cross-sectional data from 2005 to 2010, 2013 to 2014, and 2017 to 2018. Multivariate logistic regression models were applied to assess the associations among smoking status and serum cotinine levels as well as OP and osteopenia. The relationships between serum cotinine level and OP and osteopenia were also assessed using the restricted cubic spline (RCS) method.

**Results:**

A total of 10,564 participants were included in this cross-sectional study. The mean age of the study population was 64.85 ± 9.54 years, and the patients were predominantly male (51.9%). We found that the relationships between higher serum cotinine levels (≥3 ng/ml) and the prevalence of osteoporosis (Model 1: OR=2.27 [1.91-2.69]; Model 2: OR=2.03 [1.70-2.43]; Model 3: OR=2.04 [1.70-2.45]; all *p* for trend <0.001) remained significant after adjustment for covariates by applying the lowest serum cotinine levels (<0.05 ng/ml) as the reference. Similar results were observed for current smokers, who were more likely to develop OP compared with nonsmokers (Model 1: OR=2.30 [1.90-2.79]; Model 2: OR=2.16 [1.77-2.64]; Model 3: OR=2.16 [1.77-2.65]). Moreover, higher serum cotinine levels were found to be strongly and positively correlated with the prevalence of osteopenia (OR=1.60 [1.42-1.80]). A similar relationship was observed between current smokers and the prevalence of osteopenia compared with nonsmokers (OR=1.70 [1.49-1.94]). RCS regression also showed that serum cotinine levels were nonlinearly and positively correlated with OP and osteopenia, with inflection points of 5.82 ng/ml and 3.26 ng/ml, respectively.

**Conclusion:**

This study showed that being a smoker was associated with the prevalence of OP or osteopenia compared with being a nonsmoker and that there was a strong nonlinear positive dose−response relationship between serum cotinine levels and OP and osteopenia.

## Introduction

Osteoporosis (OP) is a systemic bone disorder characterized by low bone mineral density (BMD) and skeletal fragility, which increases the risk of fracture (1–3). It is the most widespread metabolic bone disease worldwide and a crucial source of morbidity and mortality (4, 5).

Studies have shown that subjects who are actively and passively exposed to tobacco are at an elevated risk for multiple health conditions, including osteopenia and an increased risk of osteoporotic fractures (6–8). Cigarette smoke contains a multitude of toxic and harmful substances, such as nicotine, heavy metals (arsenic, cadmium and lead), and tar, which may alter the skeletal system and reduce bone density (9–12). Cotinine, a significant proximal metabolite of nicotine, is regarded as a trustworthy and sensitive indicator of tobacco smoke exposure within the past 72-hours. As a result, cotinine is currently recognized as a distinctive chemical reflecting an individual’s degree of tobacco smoke exposure (13–15). According to a new study, exposure to cigarette smoke induces oxidative stress by increasing superoxide radicals and decreasing intracellular glutathione in MSCs, which adversely affects osteogenic differentiation (16). Cessation of smoking led to increases in serum levels of osteocalcin and uncarboxylated osteocalcin and BMD in humans (17).

However, clinical data on the associations between tobacco smoke exposure and the prevalence of OP or osteopenia are scarce. The goal of this study was to demonstrate an association between serum cotinine levels and self-reported smoking status with the prevalence of OP and osteopenia in a large national sample using NHANES data from 2005 to 2010, 2013 to 2014, and 2017 to 2018. Our findings provide epidemiological evidence to further investigate the associations of tobacco smoke exposure with OP and osteopenia.

## Methods

### Study population and design

The NHANES is a study project that aims to assess the health and nutritional status of American adults and children (18). There are demographic, socioeconomic, nutritional, and health-related questions in the NHANES interviews. The National Center for Health Statistics (NCHS) Research Ethics Review Board approved the NHANES research protocols, and all participants provided written informed consent.

We looked at NHANES descriptive data from 2005–2010, 2013–2014, and 2017–2018. Participants aged ≥50 years were enrolled, and those with missing serum cotinine and bone mineral density (BMD) data were excluded.

### Assessment of tobacco exposure

During the household interview, adults aged 20 and up self-reported their smoking status. Participants who claimed to have smoked fewer than 100 cigarettes in their lives were labeled ‘never smokers’. Former smokers were individuals who had smoked more than 100 cigarettes in their lives but had quit, while current smokers were those who were currently smoking.

Cotinine is a primary nicotine metabolite used as a marker of active smoking and as an indicator of exposure to secondhand smoke (19). Serum cotinine was determined by isotope dilution-high-performance liquid chromatography/atmospheric pressure chemical ionization tandem mass spectrometry (20). As in previous investigations (21), those below the lower detection limit were considered unexposed. We generated cotinine categories representing smoking exposure and utilized the newly recommended cut-off point of 3 ng/ml by Benowitz et al. (22) to separate smokers from nonsmokers. Cotinine levels were ranked as follows: cotinine <0.05 ng/ml, cotinine 0.05–2.99 ng/ml, and cotinine ≥3 ng/ml.

### BMD measurements and definition of osteopenia and osteoporosis

BMD (measured in grams/cm^2^) was evaluated using a dual X-ray absorptiometry technique (QDR 4500A fan-beam densitometers [Hologic Inc]) while the participants visited mobile examination centers. The left hip (or right hip, in case of left hip replacement or metal object injection) was routinely scanned to report total BMD of the femur, femoral neck, and trochanter. The exclusion criteria for assessing participants’ dual X-ray absorptiometry followed those of the NHANES recommendations.

The WHO criteria for osteopenia and osteoporosis (23) identify low bone mineral density for male and female individuals aged 50 years and older. This method uses the BMD data from young male and female individuals as threshold values. Male and female individuals aged between 20 and 29 years were selected as the reference group in the current study since prospective data demonstrated femur bone loss in female participants in their thirties (24). Osteopenia was defined as a BMD value that was between 1 and 2.5 standard deviations (SDs) below the mean of male and female participants aged between 20 and 29 years; osteoporosis was defined as a BMD value of more than 2.5 SD below the young reference mean. Both of these conditions were considered to have low bone density. This criteria has been applied to each region of interest.

### Covariates

In the NHANES, data were collected using a standard participant questionnaire administered throughout a household interview, along with a medical assessment for each participant. The covariates considered in this study included age, sex, race, education level, poverty, drinking status, physical activity, energy intake level, total cholesterol (TC), high-density lipoprotein cholesterol (HDL-C), serum calcium, history of prednisone or cortisone, self-reported diabetes, self-reported hypertension, self-reported cardiovascular disease (CVD), and self-reported cancer. Poverty was assessed using the poverty income ratio (PIR) and was defined as a PIR of 1 for a particular family. For drinking status, participants were categorized as being nondrinkers, low-to-moderate drinkers (<2 drinks/day in men and <1 drink/day in women), or heavy drinkers (≥2 drinks/day in men and ≥1 drink/day in women). The intake of energy was calculated by averaging the two values for the two 24-hour recall interviews. In line with their physical activity levels, the participants were classified as active, insufficiently active, and inactive (25). Descriptions of each variable are presented in https://www.cdc.gov/Nchs/Nhanes/continuousnhanes/.

### Statistical analysis

Continuous variables were reported as the means (standard deviations) or medians (interquartile ranges) and compared by adopting Student’s t test (normal distribution) or the Mann−Whitney U test (nonnormal distribution). By adopting the chi-square test, categorical variables were represented as absolute values (percentages) and compared. As continuous variables, cotinine levels were log2-transformed to achieve a normal distribution. The “mice” package utilized the random forest algorithm for multiple interpolation of the missing data. All statistical analyses were conducted by utilizing R Statistical Software, version 4.2.0, and results with *p* values < 0.05 (two-sided) were considered statistically significant.

The connections between tobacco exposure and the prevalence of osteoporosis as well as osteopenia were investigated by adopting three consecutive multivariate logistic regression models. Model 1 was adjusted for age (continuous), sex (male or female), and race/ethnicity (Mexican American, Other Hispanic, Non-Hispanic White, Non-Hispanic Black, or other race). Model 2 was adjusted for Model 1 by adding education level (below high school, high school, or above high school), drinking status (nondrinker, low-to-moderate drinker, or heavy drinker), family income-poverty ratio (<1.0, or ≥1.0), physical activity (inactive, insufficiently active, active), and total energy intake (log2-transformed). Model 3 was based on Model 2 and included additional adjustments for TC (continuous), HDL-C (continuous), serum calcium (continuous), history of prednisone or cortisone (yes or no), self-reported hypertension (yes or no), self-reported diabetes (yes or no), self-reported cardiovascular disease (yes or no), and self-reported cancer (yes or no). Furthermore, restricted cubic spline (RCS) with three knots (10th, 50th, and 90th percentiles) was adopted to study dose−response associations. Nonlinearity was examined by analysis of variance (ANOVA). By adopting segmented regression, the threshold inflection of linearity was computed to fit the piecewise-linear relationship between tobacco exposure and the prevalence of osteoporosis and osteopenia.

## Results

### Characteristics of the study participants

**Figure 1:**
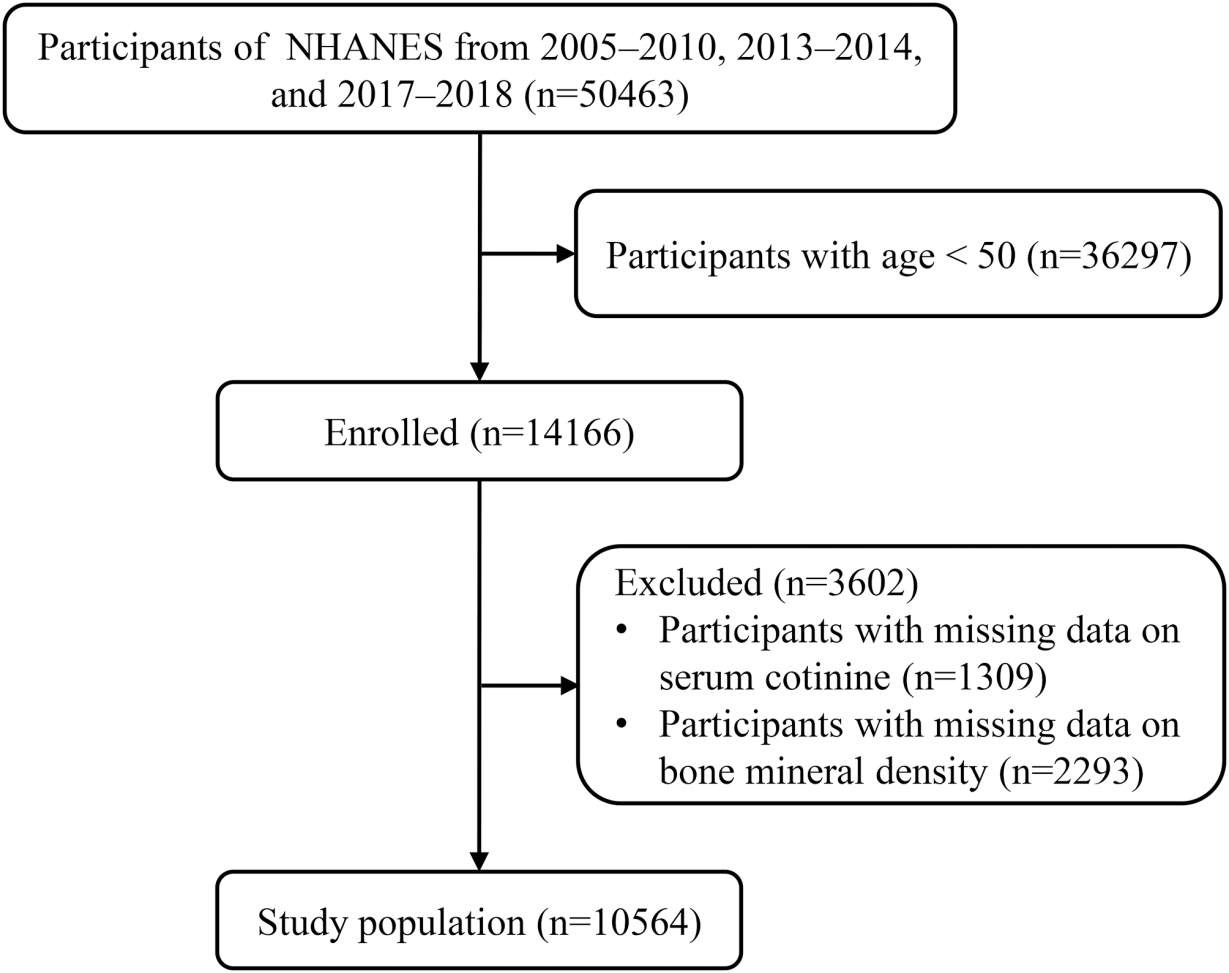
The flow chart of selection of included studies.

A total of 50463 participants from NHANES 2005–2010, 2013–2014 and 2017–2018 were included. Of these, those aged < 50 (n=36297) and those with missing data on serum cotinine (n=1309) and BMD (n=2293) were excluded. In total, 10564 eligible participants were enrolled (**Figure 1**). **Table S1** presents the baseline characteristics of participants according to NHANES 2005-2010, 2013-2014, and 2017-2018.

In this study, the thresholds for osteoporosis in men were 0.73 gm/cm^2^ or less for the total femur, 0.61 gm/cm^2^ or less for the femoral neck, 0.50 gm/cm^2^ or less for the trochanter, and 0.86 gm/cm^2^ or less for the intertrochanter; among female smokers, the thresholds were 0.65 gm/cm^2^ or less for the total femur, 0.56 gm/cm^2^ or less for the femoral neck, 0.44 gm/cm^2^ or less for the trochanter, and 0.77 gm/cm^2^ or less for the intertrochanter. The cutoff values for osteopenia in men were 0.73 to 0.94 gm/cm^2^, 0.61 to 0.83 gm/cm^2^, 0.50 to 0.69 gm/cm^2^ and 0.86 to 1.11 gm/cm^2^ for the total femur, femoral neck, femoral rotor and intertrochanter, respectively; for women, they were 0.65 to 0.84 gm/cm^2^, 0.56 to 0.76 gm/cm^2^, 0.44 to 0.61 gm/cm^2^ and 0.77 to 0.99 gm/cm^2^ for the total femur, femoral neck, femoral rotor and intertrochanter, respectively (**Table 1**). Moreover, the overall mean age of the reference group (men or women aged 20 to 29 years) was 24.45 ± 2.91 years (**Table S2**). A total of 797 (36.0%) participants had cotinine levels ≥3 ng/ml, and 663 (30.0%) participants in the reference group were current smokers. The proportion of cotinine levels was significantly higher in men than in women.

**Table 1:** Mean femoral bone mineral density (BMD) of 20–29-year-old men and women in NHANES 2005–2010, 2013–2014, and 2017–2018.

Region of interest	Mean (gm/cm^2^)	SD (gm/cm^2^)	BMD cutoff values for	
			Osteopenia	Osteoporosis
Men (n=1149)				
Total femur	1.09	0.15	0.73-0.94	<0.73
Femoral neck	0.97	0.14	0.61-0.83	<0.61
Trochanter	0.82	0.13	0.50-0.69	<0.50
Intertrochanter	1.28	0.17	0.86-1.11	<0.86
Women (n=1062)				
Total femur	0.97	0.13	0.65-0.84	<0.65
Femoral neck	0.89	0.13	0.56-0.76	<0.56
Trochanter	0.72	0.11	0.44-0.61	<0.44
Intertrochanter	1.14	0.15	0.77-0.99	<0.77

For each of the four regions of interest, low bone density was defined as: (1) osteopenia: a BMD value between 1 standard deviation (SD) and 2.5 SD below the mean of men or women age 20–29 years; and (2) osteoporosis: a BMD value >2.5 SD below the young reference mean.

The characteristics of the enrolled individuals are presented in **Table 2**. The mean age of the study population was 64.85 ± 9.54 years, and the patients were predominantly male (51.9%). By applying cotinine levels, 2228 (21.1%) participants had cotinine levels ≥3 ng/ml, 2179 (20.6%) participants had cotinine levels between 0.05 and 2.99 ng/ml, and 6157 (58.3%) participants had cotinine levels <0.05 ng/ml. The self-reported smoking status revealed that there were 1747 (16.5%) current smokers, 3612 (34.2%) former smokers, and 5205 (49.3%) nonsmokers among the participants. Participants with cotinine level ≥3 ng/ml and self-reported smokers were more likely to be younger, male, and non-Hispanic Black, to have lower education, to have poverty, to be a drinker, to have a low level of physical activity, to have a history of prednisone or cortisone use, and to have more comorbidity than those with a cotinine level <0.05 ng/ml and those who were self-reported nonsmokers.

**Table 2:** Baseline characteristics of participants in NHANES 2005–2010, 2013–2014, and 2017–2018.

Characteristics	Total	Cotinine category, %			*P* value	Self-reported smoking status, %			*P* value
		<0.05 ng/mL	0.05–2.99 ng/mL	≥3 ng/mL		Nonsmoker	Former smoker	Current smoker	
Participants, n	10564	6157	2179	2228		5205	3612	1747	
Age, years	64.85 (9.54)	66.00 (9.71)	64.95 (9.44)	61.59 (8.37)	<0.001	64.81 (9.74)	66.84 (9.31)	60.86 (8.05)	<0.001
Male, %	5478 (51.9)	2880 (46.8)	1190 (54.6)	1408 (63.2)	<0.001	2069 (39.8)	2363 (65.4)	1046 (59.9)	<0.001
Race/ethnicity, %					<0.001				<0.001
Mexican American	1473 (13.9)	1009 (16.4)	241 (11.1)	223 (10.0)		799 (15.4)	464 (12.8)	210 (12.0)	
Other Hispanic	913 (8.6)	625 (10.2)	160 (7.3)	128 (5.7)		521 (10.0)	278 (7.7)	114 (6.5)	
Non-Hispanic White	5262 (49.8)	3159 (51.3)	1017 (46.7)	1086 (48.7)		2386 (45.8)	2063 (57.1)	813 (46.5)	
Non-Hispanic Black	2033 (19.2)	803 (13.0)	574 (26.3)	656 (29.4)		932 (17.9)	594 (16.4)	507 (29.0)	
Other race	883 (8.4)	561 (9.1)	187 (8.6)	135 (6.1)		567 (10.9)	213 (5.9)	103 (5.9)	
Education level, %					<0.001				<0.001
Below high school	2959 (28.0)	1444 (23.5)	700 (32.1)	815 (36.6)		1323 (25.4)	979 (27.1)	657 (37.6)	
High school	2542 (24.1)	1334 (21.7)	586 (26.9)	622 (27.9)		1182 (22.7)	877 (24.3)	483 (27.6)	
Above high school	5063 (47.9)	3379 (54.9)	893 (41.0)	791 (35.5)		2700 (51.9)	1756 (48.6)	607 (34.7)	
Poverty, %	1624 (15.4)	627 (10.2)	398 (18.3)	599 (26.9)	<0.001	676 (13.0)	456 (12.6)	492 (28.2)	<0.001
Drinking status, %					<0.001				<0.001
Nondrinker	2501 (23.7)	1635 (26.6)	581 (26.7)	285 (12.8)		1826 (35.1)	466 (12.9)	209 (12.0)	
Low-to-moderate drinker	7397 (70.0)	4230 (68.7)	1484 (68.1)	1683 (75.5)		3196 (61.4)	2884 (79.8)	1317 (75.4)	
Heavy drinker	666 (6.3)	292 (4.7)	114 (5.2)	260 (11.7)		183 (3.5)	262 (7.3)	221 (12.7)	
Physical activity, %					<0.001				<0.001
Inactive	3276 (31.0)	1783 (29.0)	728 (33.4)	765 (34.3)		1597 (30.7)	1073 (29.7)	606 (34.7)	
Insufficiently active	3605 (34.1)	2185 (35.5)	733 (33.6)	687 (30.8)		1761 (33.8)	1304 (36.1)	540 (30.9)	
Active	3683 (34.9)	2189 (35.6)	718 (33.0)	776 (34.8)		1847 (35.5)	1235 (34.2)	601 (34.4)	
Energy intake, kcal/day	1776.75 [1371.00, 2285.00]	1757.50 [1371.00, 2226.00]	1759.50 [1344.50, 2290.50]	1852.75 [1404.00, 2437.62]	<0.001	1702.00 [1328.00, 2184.00]	1841.75 [1425.38, 2342.12]	1859.50 [1409.25, 2450.00]	<0.001
Total cholesterol, mg/dL	197.73 (42.93)	197.86 (42.20)	196.70 (43.49)	198.40 (44.34)	0.396	200.24 (42.05)	193.28 (43.49)	199.48 (43.64)	<0.001
HDL-C, mmol/L	1.40 (0.43)	1.42 (0.42)	1.38 (0.41)	1.36 (0.45)	<0.001	1.44 (0.42)	1.36 (0.41)	1.37 (0.46)	<0.001
Serum calcium, mg/dL	9.43 (0.38)	9.44 (0.38)	9.42 (0.37)	9.44 (0.39)	0.112	9.43 (0.38)	9.43 (0.38)	9.44 (0.39)	0.364
History of prednisone or cortisone, %	651 (6.2)	345 (5.6)	142 (6.5)	164 (7.4)	0.009	288 (5.5)	235 (6.5)	128 (7.3)	0.015
Hypertension, %	5551 (52.5)	3264 (53.0)	1199 (55.0)	1088 (48.8)	<0.001	2729 (52.4)	2002 (55.4)	820 (46.9)	<0.001
Diabetes, %	1990 (18.8)	1182 (19.2)	443 (20.3)	365 (16.4)	0.002	974 (18.7)	749 (20.7)	267 (15.3)	<0.001
CVD, %	1919 (18.2)	1016 (16.5)	446 (20.5)	457 (20.5)	<0.001	765 (14.7)	810 (22.4)	344 (19.7)	<0.001
Cancer, %	1696 (16.1)	1100 (17.9)	313 (14.4)	283 (12.7)	<0.001	787 (15.1)	701 (19.4)	208 (11.9)	<0.001
BMD, gm/cm^2^									
Total femur	0.92 (0.16)	0.92 (0.16)	0.94 (0.17)	0.92 (0.17)	<0.001	0.91 (0.16)	0.94 (0.16)	0.91 (0.17)	<0.001
Femoral neck	0.77 (0.14)	0.76 (0.14)	0.78 (0.15)	0.77 (0.14)	<0.001	0.76 (0.15)	0.77 (0.14)	0.77 (0.14)	<0.001
Trochanter	0.70 (0.14)	0.70 (0.14)	0.71 (0.15)	0.69 (0.14)	<0.001	0.69 (0.14)	0.72 (0.14)	0.69 (0.14)	<0.001
Intertrochanter	1.10 (0.20)	1.09 (0.19)	1.12 (0.20)	1.10 (0.20)	<0.001	1.09 (0.20)	1.12 (0.19)	1.09 (0.20)	<0.001
Osteoporosis, %	1106 (10.5)	657 (10.7)	190 (8.7)	259 (11.6)	0.005	546 (10.5)	356 (9.9)	204 (11.7)	0.124
Osteopenia, %	6927 (65.6)	4105 (66.7)	1330 (61.0)	1492 (67.0)	<0.001	3373 (64.8)	2375 (65.8)	1179 (67.5)	0.119

Normally distributed continuous variables are described as means ± SEs, and continuous variables without a normal distribution are presented as medians [interquartile ranges]. Categorical variables are presented as numbers (percentages). HDL-C, high-density lipoprotein cholesterol; CVD, cardiovascular disease; BMD, bone mineral density.

### Associations between serum cotinine levels and smoking status with the prevalence of osteoporosis

**Table 3** displays the associations between serum cotinine levels and the prevalence of osteoporosis in both continuous and categorical analyses. Regardless of adjustment for covariates, the continuous analysis revealed that log2-transformed cotinine levels showed a noticeable positive association with the prevalence of osteoporosis. The categorical analysis indicated that the association between higher serum cotinine levels (≥3 ng/ml) and the prevalence of osteoporosis (Model 1: OR=2.27 [1.91-2.69]; Model 2: OR=2.03 [1.70-2.43]; Model 3: OR=2.04 [1.70-2.45]; all *p* for trend <0.001) remained significant after adjustment for covariates using the lowest serum cotinine levels (<0.05 ng/ml) as a reference. Similar results were observed in that current smokers were highly associated with the prevalence of osteoporosis compared with nonsmokers (Model 1: OR=2.30 [1.90-2.79]; Model 2: OR=2.16 [1.77-2.64]; Model 3: OR=2.16 [1.77-2.65]; all *p* for trend <0.001).

**Table 3:** Association of smoking status, serum cotinine level and the prevalence of osteoporosis.

	OR (95% CI)		
	Model 1	Model 2	Model 3
Log2-transformed cotinine, ng/mL	1.07 (1.05-1.08)	1.06 (1.04-1.07)	1.06 (1.04-1.07)
Cotinine categories			
<0.05 ng/mL	1.00 [Reference]	1.00 [Reference]	1.00 [Reference]
0.05–2.99 ng/mL	0.99 (0.83-1.18)	0.91 (0.76-1.09)	0.91 (0.76-1.10)
≥3 ng/mL	2.27 (1.91-2.69)	2.03 (1.70-2.43)	2.04 (1.70-2.45)
*P* for trend	<0.001	<0.001	<0.001
Self-reported smoking status, %			
Nonsmokers	1.00 [Reference]	1.00 [Reference]	1.00 [Reference]
Former smokers	0.96 (0.82-1.12)	1.01 (0.86-1.18)	1.02 (0.87-1.20)
Current smokers	2.30 (1.90-2.79)	2.16 (1.77-2.64)	2.16 (1.77-2.65)
*P* for trend	<0.001	<0.001	<0.001

Model 1 was adjusted as age, sex, and race;

Model 2 was adjusted as model 1 plus education level, drinking status, poverty, physical activity, and total energy intake.

Model 3 was adjusted as model 2 plus total cholesterol, high-density lipoprotein cholesterol, serum calcium, history of prednisone or cortisone, hypertension, diabetes, cardiovascular disease, and cancer.

### Associations between serum cotinine levels and smoking status with the prevalence of osteopenia

Further examination of the associations between serum cotinine levels, smoking status, and the prevalence of osteopenia is presented in **Table 4**. As illustrated by the continuous analysis, after adjustment for covariates, there was a markedly positive relationship between log2-transformed serum cotinine levels and the prevalence of osteopenia. In Model 3, the categorical analysis revealed that the multivariate odds ratios (95% confidence intervals [CI]) for osteopenia increased monotonically to 0.95 (0.85-1.06) and 1.60 (1.42-1.80) (*p* for trend < 0.001) with higher serum cotinine levels (≥3 ng/ml). Similar relationships were observed between current smokers and the prevalence of osteopenia compared with nonsmokers (OR=1.70 [1.49-1.94]; *p* for trend < 0.001).

**Table 4:** Association of smoking status, serum cotinine level and the prevalence of osteopenia.

	OR (95% CI)		
	Model 1	Model 2	Model 3
Log2-transformed cotinine, ng/mL	1.04 (1.03-1.05)	1.04 (1.03-1.05)	1.04 (1.03-1.05)
Cotinine categories			
<0.05 ng/mL	1.00 [Reference]	1.00 [Reference]	1.00 [Reference]
0.05–2.99 ng/mL	0.96 (0.86-1.07)	0.94 (0.84-1.04)	0.95 (0.85-1.06)
≥3 ng/mL	1.65 (1.47-1.85)	1.59 (1.41-1.79)	1.60 (1.42-1.80)
*P* for trend	<0.001	<0.001	<0.001
Self-reported smoking status, %			
Nonsmokers	1.00 [Reference]	1.00 [Reference]	1.00 [Reference]
Former smokers	0.99 (0.90-1.09)	1.01 (0.92-1.12)	1.04 (0.94-1.15)
Current smokers	1.72 (1.52-1.95)	1.69 (1.49-1.93)	1.70 (1.49-1.94)
*P* for trend	<0.001	<0.001	<0.001

Model 1 was adjusted as age, sex, and race;

Model 2 was adjusted as model 1 plus education level, drinking status, poverty, physical activity, and total energy intake.

Model 3 was adjusted as model 2 plus total cholesterol, high-density lipoprotein cholesterol, serum calcium, history of prednisone or cortisone, hypertension, diabetes, cardiovascular disease, and cancer.

### Nonlinear associations between serum cotinine levels and the prevalence of osteoporosis and osteopenia

**Figure 2:**
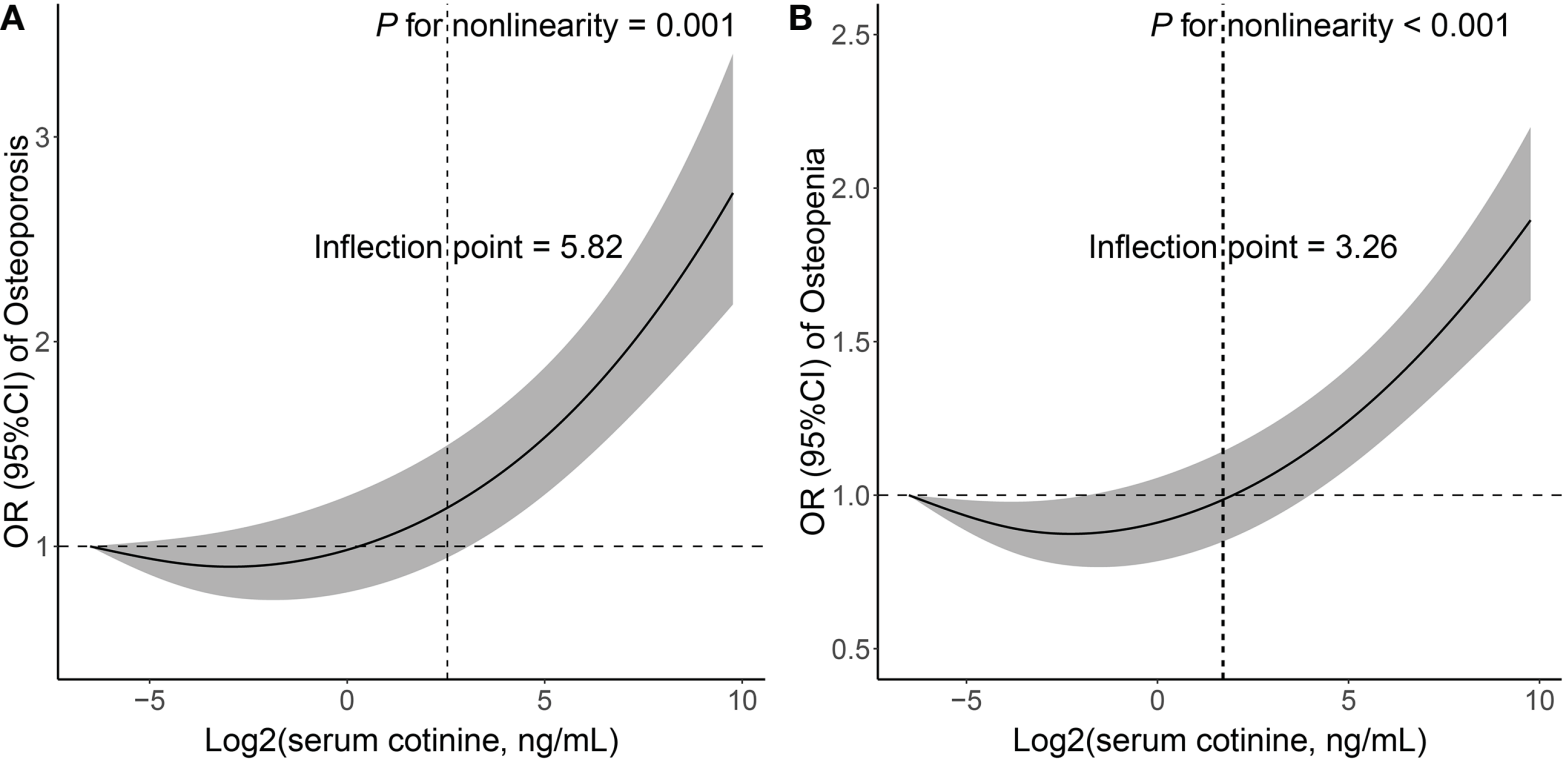
Associations between log2-transformed serum cotinine levels and the prevalence of osteoporosis **(A)** as well as osteopenia **(B)**.

RCS regression with multivariable-adjusted associations was adopted to demonstrate dose−response associations between log2-transformed serum cotinine levels and the prevalence of osteoporosis as well as osteopenia (**Figure 2**). Serum cotinine levels were nonlinearly and positively correlated with the prevalence of osteoporosis (*p* for nonlinearity = 0.001) and osteopenia (*p* for nonlinearity < 0.001), with inflection points of 5.82 ng/ml and 3.26 ng/ml, respectively.

## Discussion

As shown by our findings, both serum cotinine levels and self-reported smoking status have an impact on OP and osteopenia. This relationship remained constant even after the addition of other factors (education level, drinking status, poverty status, physical activity status, total energy intake, total cholesterol level, high-density lipoprotein cholesterol level, serum calcium level, history of prednisone or cortisone use, and diagnoses of hypertension, diabetes, and cardiovascular disease, and cancer). Our dose−response analysis also showed nonlinear and positive associations between serum cotinine and the prevalence of OP as well as osteopenia.

A 2011-2018 NHANES cross-sectional study showed that elevated serum cotinine levels were associated with reduced lumbar BMD in 7905 participants aged 30 years and over, particularly in women (26). This study demonstrated that reducing cigarette exposure and maintaining serum cotinine at lower levels may be beneficial for bone health in adults. However, the study failed to calculate the specific breakpoints of the curve. Second, other confounding factors were not considered, so there is a possibility of bias. Finally, the sample size included was insufficient. Therefore, we further analyzed the NHANES cross-sectional study from 2005-2018, 10,564 participants aged ≥50 years, to assess not only the association between serum cotinine levels with OP and osteopenia, but also the association between smoking status with OP and osteopenia. In addition, we considered the effect of other confounders on the study results, such as (drinking status, poverty status, total energy intake, total cholesterol level, high-density lipoprotein cholesterol level, history of prednisone or cortisone use, and diagnoses of hypertension, diabetes, and cardiovascular disease, and cancer), reducing the possibility of biased results. In last, we analyzed the relationship between serum cotinine levels and OP and osteopenia, which were nonlinearly and positively correlated, with inflection points of 5.82 ng/ml and 3.26 ng/ml, respectively. This provides a preliminary basis for the study of OP or osteopenia and the mechanism of action of serum cotinine.

We found that associations between smoking and OP and osteopenia were consistent with other cross-sectional studies (27–29). For example, the results of a Swedish study investigating the association between smoking status and skeletal parameters (area BMD, volume BMD, etc.) in 1068 young (mean age 18.9 years) men showed that smokers had lower whole-body (-2.1%), lumbar spine (-4.3%), femoral neck (- 5.3%) and femoral rotor (-6.6%) BMD, but there was no difference in the distribution of volumetric BMD between smokers and nonsmokers (27). In addition, an Icelandic quantitative computed tomography scan of the hip including 2673 older adults (55.9% female) aged 66 to 92 years at baseline demonstrated that the volumetric BMD of cortical bone in the hip was lower in current smokers than in those who were never smokers and that the volumetric BMD of whole hip, volumetric BMD of trabecular bone, and volumetric BMD of cortical bone were lower in current smokers than in nonsmokers. The lower proportions of total volumetric BMD, trabecular bone volumetric BMD, and cortical bone volumetric BMD among current smokers suggest that smoking may accelerate aging-induced osteopenia (28). As reported by relevant studies, male smokers had lower trabecular bone mineral density (-26.6 to -30.3%), and lower trabecular bone scores (-13.5 to -15.3%) in the radius and tibia than current quitters or those who were never smokers. There were fewer trabeculae, thinner areas, and larger intertrabecular spaces, but there was no significant variability in cortical bone parameters. Among female smokers, radial cortical porosity was twice as large in smokers as in nonsmokers, and tibial cortical porosity was 50% larger in smokers than in nonsmokers, with no statistically significant differences in trabecular parameters between smokers and nonsmokers (29).

As revealed by our in-depth research on the nonlinear and positive correlation of serum cotinine levels with OP and osteopenia, the relationship between smoking and osteopenia is at least partially correlated with the effects of nicotine. Indeed, studies have demonstrated that nicotine not only has a direct toxic effect on osteoblasts but is also associated with an increase in osteoclasts (9, 30). In addition, nicotine inhibits aromatase activity and exerts antiestrogenic effects, and the decrease in gonadal hormone levels gives rise to a decrease in osteoblast activity and proliferation and an increase in the resorptive activity of osteoclasts (31, 32). Finally, nicotine heightens inflammation, disrupts the body’s oxidative-antioxidant balance, elevates malondialdehyde levels, and decreases superoxide dismutase and catalase levels, all of which may contribute to osteoporosis (16, 33).

NHANES data were collected and screened by adopting a standardized, uniform protocol to ensure the accuracy, consistency, and reliability of the study data and results. The large community sample ensured the reliability of the results. The inclusion of some important confounding factors in the regression analysis was more intuitive and comprehensive than the mechanism study. Nevertheless, the study still has some limitations. Above all, because this study was based on a cross-sectional survey of NHANES, causal relationships among dependent, independent, and covariate variables could not be inferred. Moreover, because the half-life of nicotine varies among and within individuals, a single indicator of cotinine may not necessarily reflect long-term nicotine exposure. Last but not least, NHANES data sources are measured or collected only once, which heightens the potential for data bias. Accordingly, the database may be replicated multiple times in subsequent studies.

In summary, our study demonstrated that tobacco smoke exposure was correlated with the prevalence of OP or osteopenia in a representative sample of the elderly population in the U.S. The dose-effect phenomenon exhibited a nonlinear and positive relationship between serum cotinine levels and the prevalence of OP or osteopenia. Consequently, the mechanism of OP or osteopenia and serum cotinine needs to be further explored in the future.

## Data availability statement

The original contributions presented in the study are included in the article/**Supplementary Material**. Further inquiries can be directed to the corresponding author.

## Ethics statement

The studies involving human participants were reviewed and approved by The National Center for Health Statistics (NCHS) Research Ethics Review Board. The patients/participants provided their written informed consent to participate in this study.

## Author contributions

WH and SC designed the study, analyzed the data, and wrote the manuscript. CZ, LZ and YG analyzed the data. ZZ and LZ revised the manuscript. WH and SC have contributed equally to this work. All authors contributed to the article and approved the submitted version.
