# Supplementary material for: Associations between smoke exposure and osteoporosis or osteopenia in a US NHANES population of elderly individuals
Source: Front Endocrinol (Lausanne). 2023 Feb 3;14:1074574. doi: 10.3389/fendo.2023.1074574 (PMC9935577; doi:10.3389/fendo.2023.1074574)
Supplement: Supplementary file 1 [file Table_1.docx]

**Online Supplementary Material**

**Associations between smoke exposure and osteoporosis or osteopenia in a US NHANES population of elderly individuals**

Wenyuan Hou, Shaoqi Chen, Caiyu Zhu, Yifan Gu, Lei Zhu, Zhengxin Zhou

**Table S1.** Baseline characteristics of participants in NHANES 2005–2010, 2013–2014, and 2017–2018.

| Characteristics | 2005–2010 | 2013–2014 | 2017–2018 |
| --- | --- | --- | --- |
| Participants, n | 6212 | 2154 | 2198 |
| Age, years | 65.16 (9.81) | 64.15 (9.22) | 64.67 (9.04) |
| Male, % | 3260 (52.5) | 1069 (49.6) | 1149 (52.3) |
| Race/ethnicity, % |  |  |  |
| Mexican American | 966 (15.6) | 258 (12.0) | 249 (11.3) |
| Other Hispanic | 484 (7.8) | 189 (8.8) | 240 (10.9) |
| Non-Hispanic White | 3433 (55.3) | 1007 (46.8) | 822 (37.4) |
| Non-Hispanic Black | 1099 (17.7) | 437 (20.3) | 497 (22.6) |
| Other race | 230 (3.7) | 263 (12.2) | 390 (17.7) |
| Education level, % |  |  |  |
| Below high school | 1982 (31.9) | 519 (24.1) | 458 (20.8) |
| High school | 1500 (24.1) | 499 (23.2) | 543 (24.7) |
| Above high school | 2730 (43.9) | 1136 (52.7) | 1197 (54.5) |
| Poverty, % | 920 (14.8) | 372 (17.3) | 332 (15.1) |
| Drinking status, % |  |  |  |
| Nondrinker | 1535 (24.7) | 518 (24.0) | 448 (20.4) |
| Low-to-moderate drinker | 4242 (68.3) | 1491 (69.2) | 1664 (75.7) |
| Heavy drinker | 435 (7.0) | 145 (6.7) | 86 (3.9) |
| Physical activity, % |  |  |  |
| Inactive | 1983 (31.9) | 640 (29.7) | 653 (29.7) |
| Insufficiently active | 2167 (34.9) | 739 (34.3) | 699 (31.8) |
| Active | 2062 (33.2) | 775 (36.0) | 846 (38.5) |
| Energy intake, kcal/day | 1764.75 [1363.38, 2252.12] | 1786.00 [1374.12, 2301.38] | 1810.00 [1392.62, 2384.88] |
| Total cholesterol, mg/dL | 201.09 (42.67) | 193.33 (41.80) | 192.57 (43.85) |
| HDL-C, mmol/L | 1.40 (0.43) | 1.41 (0.44) | 1.41 (0.41) |
| Serum calcium, mg/dL | 9.46 (0.38) | 9.46 (0.36) | 9.33 (0.38) |
| History of prednisone or cortisone, % | 373 (6.0) | 122 (5.7) | 156 (7.1) |
| Hypertension, % | 3203 (51.6) | 1183 (54.9) | 1165 (53.0) |
| Diabetes, % | 1074 (17.3) | 408 (18.9) | 508 (23.1) |
| CVD, % | 1156 (18.6) | 356 (16.5) | 407 (18.5) |
| Cancer, % | 1007 (16.2) | 338 (15.7) | 351 (16.0) |
| BMD, gm/cm^2^ |  |  |  |
| Total femur | 0.92 (0.17) | 0.93 (0.16) | 0.92 (0.16) |
| Femoral neck | 0.77 (0.14) | 0.76 (0.14) | 0.76 (0.14) |
| Trochanter | 0.70 (0.14) | 0.70 (0.13) | 0.70 (0.13) |
| Intertrochanter | 1.09 (0.20) | 1.11 (0.19) | 1.10 (0.19) |
| Osteoporosis, % | 642 (10.3) | 222 (10.3) | 242 (11.0) |
| Osteopenia, % | 4008 (64.5) | 1446 (67.1) | 1473 (67.0) |
| Cotinine category, % |  |  |  |
| <0.05 ng/mL | 3422 (55.1) | 1344 (62.4) | 1391 (63.3) |
| 0.05–2.99 ng/mL | 1467 (23.6) | 343 (15.9) | 369 (16.8) |
| ≥3 ng/mL | 1323 (21.3) | 467 (21.7) | 438 (19.9) |
| Self-reported smoking status, % |  |  |  |
| Nonsmoker | 2915 (46.9) | 1098 (51.0) | 1192 (54.2) |
| Former smoker | 2247 (36.2) | 687 (31.9) | 678 (30.8) |
| Current smoker | 1050 (16.9) | 369 (17.1) | 328 (14.9) |

Normally distributed continuous variables are described as means ± SEs, and continuous variables without a normal distribution are presented as medians [interquartile ranges]. Categorical variables are presented as numbers (percentages). HDL-C, high-density lipoprotein cholesterol; CVD, cardiovascular disease; BMD, bone mineral density.

**Table S2.** Baseline characteristics of the reference group (men or women aged 20 to 29 years) in NHANES 2005–2010, 2013–2014, and 2017–2018.

| Characteristics | Total | Male | Female |
| --- | --- | --- | --- |
| Participants, n | 2211 | 1149 | 1062 |
| Age, years | 24.45 (2.91) | 24.43 (2.89) | 24.47 (2.94) |
| Race/ethnicity, % |  |  |  |
| Mexican American | 531 (24.0) | 298 (25.9) | 233 (21.9) |
| Other Hispanic | 211 (9.5) | 97 (8.4) | 114 (10.7) |
| Non-Hispanic White | 903 (40.8) | 452 (39.3) | 451 (42.5) |
| Non-Hispanic Black | 452 (20.4) | 243 (21.1) | 209 (19.7) |
| Other race | 114 (5.2) | 59 (5.1) | 55 (5.2) |
| Cotinine category, % |  |  |  |
| <0.05 ng/mL | 660 (29.9) | 275 (23.9) | 385 (36.3) |
| 0.05–2.99 ng/mL | 637 (28.8) | 312 (27.2) | 325 (30.6) |
| ≥3 ng/mL | 797 (36.0) | 497 (43.3) | 300 (28.2) |
| Missing | 117 (5.3) | 65 (5.7) | 52 (4.9) |
| Self-reported smoking status, % |  |  |  |
| Nonsmoker | 1314 (59.5) | 614 (53.4) | 700 (65.9) |
| Former smoker | 232 (10.5) | 137 (11.9) | 95 (8.9) |
| Current smoker | 663 (30.0) | 397 (34.6) | 266 (25.0) |
| Missing | 2 (0.1) | 1 (0.1) | 1 (0.1) |

Normally distributed continuous variables are described as means ± SDs. Categorical variables are presented as numbers (percentages).
